# Integrated multisectoral non-communicable disease prevention and control in China: A review of agencies and policies

**DOI:** 10.7189/jogh.10.020304

**Published:** 2020-12

**Authors:** Shiwei Liu, Zhuo Chen, Liyuan Han, Wenlan Dong, Hui Li, Jeffrey Koplan, Jing Wu, Xinhua Li

**Affiliations:** 1 National Center for Chronic and Noncommunicable Disease Control and Prevention, Chinese Center for Disease Control and Prevention, Beijing, China; 2 Tobacco Control Office, Chinese Center for Disease Control and Prevention, Beijing, China; 3 College of Public Health, University of Georgia, Athens, Georgia, USA; 4 School of Economics, University of Nottingham Ningbo China, Ningbo, Zhejiang Province, China; 5 Hwa Mei Hospital, University of Chinese Academy of Sciences, Ningbo, Zhejiang, China; 6 Department of Global Health, Ningbo Institute of Life and Health Industry, University of Chinese Academy of Sciences, Ningbo, Zhejiang, China; 7 Ningbo Municipal Center for Disease Control and Prevention, Ningbo, Zhejiang Province, China; 8 Emory Global Health Institute, Emory University, Atlanta, Georgia, USA; 9 Chinese Center for Disease Control and Prevention, Beijing, China

**Figure:**
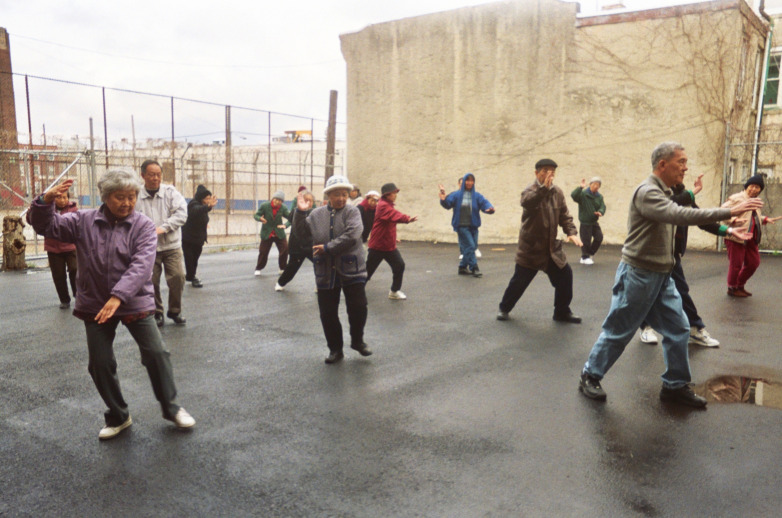
Photo: A group of Chinese seniors practicing the art of Tai Chi in order to increase their level of physical fitness (Photographer: Phyllis Nichols, available from: https://phil.cdc.gov/Details.aspx?pid=19372).

The epidemic of non-communicable diseases (NCDs) has become a global threat to human life, health, and sustainable development, particularly in low- and middle-income countries [1]. In 2017, NCDs accounted for 73.4% of all deaths worldwide and 89.5% for China [2]. The most common NCDs, eg, cardiovascular diseases (CVD), cancer, chronic respiratory diseases, and diabetes, are preventable. Mental disorders have recently been added to the list of major NCDs.

NCDs are caused by diverse and pervasive risk factors through complex causal pathways. They are social, cultural, and economic factors at the distal level, environmental and behavioral factors at the intermediate level, and metabolic and genetic determinants at the proximal level. The prevalence rates of most NCD risk factors are on the rise in China [3], posing a challenge to China’s health system. Population aging in China has exacerbated the situation with a continuing increase in the incidence of NCDs [3,4].

Given the complexities of NCDs, the World Health Organization (WHO) has called for coordinated, comprehensive, and integrated strategies to contain the NCD epidemic [5]. The integrated strategies for the NCD prevention date back to Finnish efforts begun in 1972, which sought to develop comprehensive health policies that were implemented in non-health sectors [6]. The concept has evolved, through the 1978 Alma-Ata Declaration and the 1986 Ottawa Charter, into the Health in All Policies (HiAP) in 2006 during the Finnish European Union presidency. Later updates include the Adelaide Statement and the Helsinki Statement on HiAP [7]. The UN high-level meeting on the prevention and control of NCDs in 2011 has emphasized government responsibilities and multi-sectoral actions [8]. NCD prevention and control remains a top priority of the UN, with its Resolution #68/330 of July 10, 2014, reaffirmed in 2018. These resolutions call for commitments from member country governments to reduce risk factors and improve underlying social determinants for NCDs through the implementation of interventions and policy options to facilitate health promotion.

Since the recent round of health care reform in 2009, the Chinese government has put a great deal of effort into developing a practical and actionable response to the emerging NCD epidemic with a focus on integrated strategies [9]. A recent study provides a review of the accomplishments of NCD Prevention and Control in China [10]. The objective of this viewpoint piece is to review the evolution of health agencies and policies related to NCD Prevention and Control in China divided into three stages and to discuss potential new directions.

## NCD Prevention and Control in China: Agencies and Strategies

In China, the strategy and policy framework for NCD prevention and control has evolved in three stages. The first stage extends from the establishment of the People’s Republic of China in 1949 and until 1993. In the early days of the Republic, the nascent central government had to address various epidemics, including plague, schistosomiasis, tuberculosis, polio, etc. NCDs were not a top priority during this time [11]. The MoH used its limited resources on selected NCDs/conditions in lieu of a comprehensive strategy of NCD prevention and control. The National Office for Cancer Prevention and Control was created in 1969 under the auspices of the Chinese Academy of Medical Sciences (CAMS) Cancer Hospital and was tasked with coordinating and implementing cancer prevention and control. The office was renamed the MoH Office of Cancer Prevention and Control in 1978. In 1987, the MoH established the National Office for Cardiovascular Disease Prevention and Control at CAMS Fuwai Hospital and the National Office for Cerebrovascular Disease Prevention and Control at Beijing Tiantan Hospital of Capital Medical University [12]. In addition, the MoH created an Advisory Taskforce for Dental Prevention and Control to assist the Ministry in formulating national strategies for the prevention and control of dental health issues and capacity building, coordinating academic exchange, and promoting new dental technologies and knowledge. This stage saw the development of epidemiological surveys and population-level prevention research, including the Lin-zhou Esophageal Cancer Prevention and Treatment Study [13], the Shou-gang Hypertension Study [14], and the Da-qing Diabetes Prevention Longitudinal Study [15]. Disease-specific prevention and control efforts have much success and led to the creation and development of provincial and local-level offices of disease-based prevention and control efforts.

The second stage spans from 1994-2009. During this time, China started to implement health promotion and community prevention efforts, made progress in NCD prevention and public health, and developed preliminary surveillance systems. The MoH Bureau of Epidemic Prevention was renamed to the Bureau of Disease Control and Prevention in 1994. The Division of Chronic and Non-communicable Disease Control and Prevention was established the same year. In 1996, the China Academy of Preventive Medicine (CAPM) created the Office of Chronic Disease Control and Prevention and Health Promotion. CAPM became the Chinese Center for Disease Control and Prevention (China CDC) and established the National Centre for Chronic and Non-Communicable Disease Control and Prevention (NCNCD) in 2002. The China CDC also created the Division of Chronic Disease Prevention and Community Health in 2006, which provided overarching organization and guidance for NCD prevention and control [12]. China ratified the WHO Framework Convention on Tobacco Control (FCTC) in 2003 and started to enforce FCTC on January 9, 2006. The Office of Tobacco Control moved out of the NCNCD to become a national office directly under the China CDC in 2009. The “China Healthy Lifestyles for All” initiative was launched by the MoH Bureau of Disease Control and Prevention and the China CDC in 2007 to raise awareness on preventive health and promote healthy behaviours. Over time, the public health community has seen increased attention to the goals and strategies of NCD prevention and control. However, coordinated and integrated strategies were limited to the health sector during this stage.

**Figure 1:**
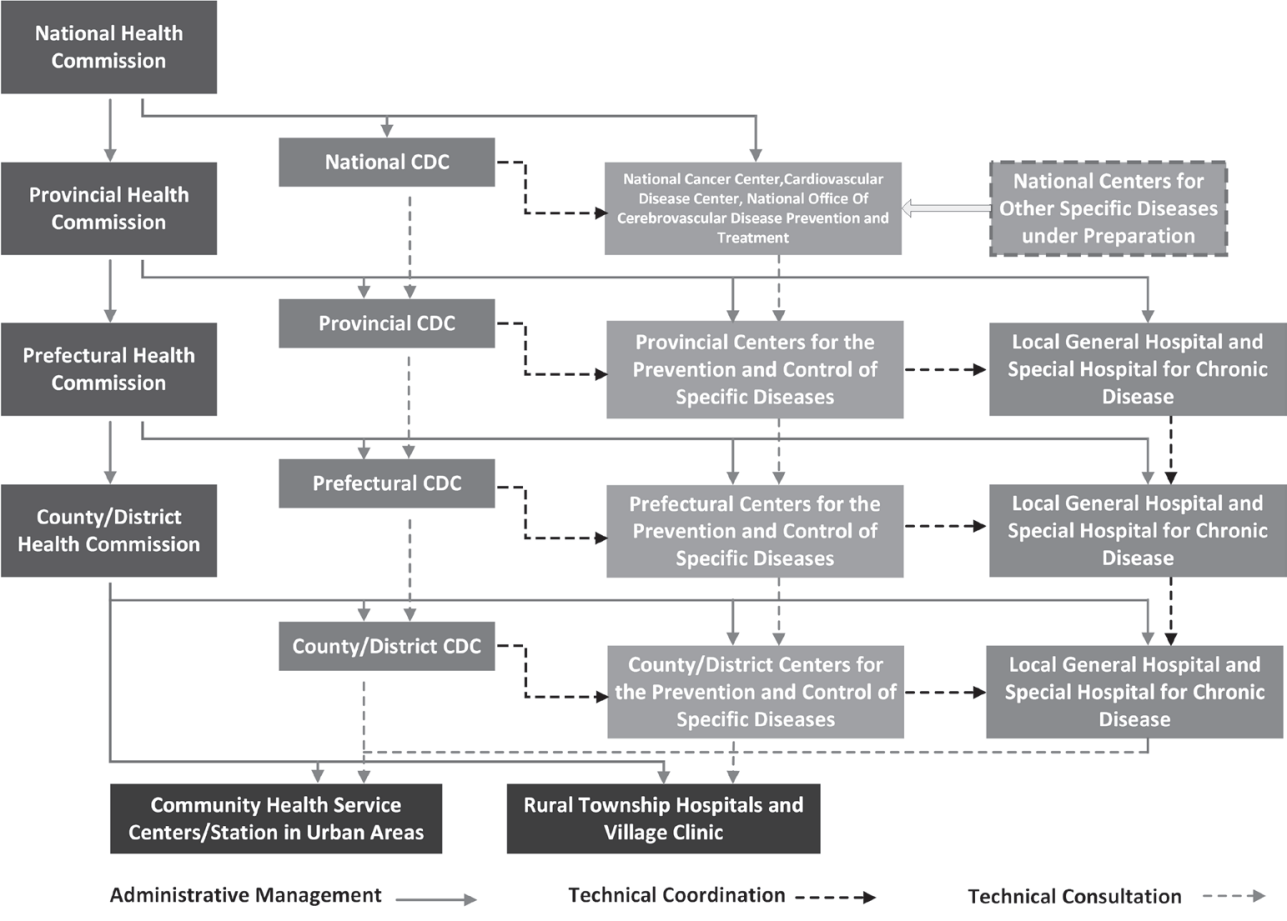
Framework of non-communicable disease prevention and control in China. CDC – Center for Disease Control and Prevention.

The third stage dates back from 2009 when China began its recent round of health reform and continues to the present day [12]. In the Circular 67: Major Tasks on the Five Priority Areas of Healthcare Reform in 2010, the National Council of China put forth five priority areas for China’s 2009 health reform: (1) expanding health care coverage to more than 90% of the population, (2) establishing a national essential medicine system, (3) improving the primary care system, (4) providing essential public health services for all, and (5) public hospital reforms. The fourth priority area is particularly relevant to NCD prevention and control. China has since developed a national NCD prevention and control system, with laudable achievements in NCD prevention and control capacity building. The non-clinical sector, especially professional/ academic organizations, has become a valuable partner. The National Cancer Center (NCC) and the National Center for Cardiovascular Diseases (NCCD) were created in 2009. The national system of NCD prevention and control consists of the Health Bureaus/Commissions and CDCs at the national, provincial, prefectural, and county levels; national centers for specific diseases or conditions (eg, cancer, CVD, etc.); and the NCD Prevention and Control Network. The Network units are affiliated with health care providers such as tertiary or secondary hospitals that have a dedicated NCD Prevention and Control unit and community/village health centers with an independent NCD Prevention and Control branch (**Figure 1**) [12]. In addition, public policies and surveillance systems have sustained development during this time, with continued growth in the investment of NCD prevention and control. NCD-related surveillances include mortality and cause of death surveillance, NCDs and risk factors surveillance survey, nutrition survey, cancer registry, CVD sentinel surveillance and epidemiological survey, chronic obstructive pulmonary disease (COPD), oral health, osteoporosis, and mental health epidemiological surveys [16]. A nationwide intervention with integrated strategies and measures has been implemented – with key initiatives, including the National Demonstration Areas Program for Integrated NCD Prevention and Control of (NCDDA) [17].

## Current Efforts of the Chinese Government on NCD Prevention and Control

Integrated NCD prevention and control has since been a top priority for the Chinese government. During 1984-2014, the Chinese government and its agencies, primarily the national government and the State Council, have issued 163 policies related to NCD prevention and control [18]. Among these health policies, Healthy China 2030 emphasized implementing integrated prevention and control strategies for NCDs by combining health policies with concerted efforts of other sectors, including transportation, energy, and environmental protection [19], in addition to strengthening the NCDDA project. Both the 13th Five-Year Plan of National Economic and Social Development and the 13th Five-Year Plan of National Hygiene and Health emphasized implementing comprehensive and integrated strategies to curb the NCD epidemics. The “China Healthy Lifestyles for All” Action Plan (2017-2025) was jointly issued by the NHC, the General Administration of Sport of China, the All-China Federation of Trade Unions, the Central Committee of the Communist Youth League, and the All-China Women’s Federation, which sends a strong signal of China’s intention to stress on multi-sectoral cooperation. China’s State Council promulgated the Circular on China’s Mid- and Long-term Plan of NCD Prevention and Treatment (2017-2025), which highlights coordinated efforts from all sectors, health education and promotion, prevention first, and effective prevention and control. This plan required 15% of all counties/districts nationwide to become national NCDDA by 2020 and 20% by 2025 [19]. A similar circular for 2012-2015 was launched by 15 Ministries and Commissions of the Chinese national government. In 2017, President Xi pronounced that China had entered a new era of NCD prevention and control with new goals. In 2019, Healthy China Action Plan, in line with Healthy China 2030, was released by China’s State Council, involving 15 actions with specific targets to be attained between 2020-30.

## Discussions

The NCD institutional development is both an indication and result of capacity building and epidemiologic transition. Recent policy developments in China have indicated a strong commitment to multi-sectoral efforts for NCD prevention and control. Sporadic efforts of institutional reform of the Chinese CDC system at provincial and prefecture-level have met criticisms that the efforts may have weakened local public health capacity. The aftermath of the COVID-19 pandemic led to calls for strengthening the disease control system among China’s public health community [20]. While such calls may have focused on infectious disease, they would have a profound impact on NCD prevention and control as well.

Another critical area of future efforts for China’s NCD prevention and control is the evaluation of existing programs and policies. While qualitative evaluations of the NCDDA exist, a systematic evaluation of the impact of NCD prevention and control programs would provide insights for future policy and program development.

## Conclusions

China’s NCD Prevention and Control Strategy and Policy framework has evolved from disease-specific prevention and control during 1949-1993, to coordinated and integrated strategies within the health sector during 1994-2009, and to integrated multisectoral NCD prevention and control since 2009. Strengthening China’s NCD prevention and control programs and its evaluation efforts are in urgent need.
